# Augmented expression of Ki-67 is correlated with clinicopathological characteristics and prognosis for lung cancer patients: an up-dated systematic review and meta-analysis with 108 studies and 14,732 patients

**DOI:** 10.1186/s12931-018-0843-7

**Published:** 2018-08-13

**Authors:** Dan-ming Wei, Wen-jie Chen, Rong-mei Meng, Na Zhao, Xiang-yu Zhang, Dan-yu Liao, Gang Chen

**Affiliations:** Department of Pathology, First Affiliated Hospital of Guangxi Medical University, Nanning, Guangxi Zhuang Autonomous Region 530021 People’s Republic of China

**Keywords:** Ki-67, Lung cancer, Meta-analysis, Prognosis, Clinicopathological characteristics

## Abstract

**Background:**

Lung cancer ranks as the leading cause of cancer-related deaths worldwide and we performed this meta-analysis to investigate eligible studies and determine the prognostic effect of Ki-67.

**Methods:**

In total, 108 studies in 95 articles with 14,732 patients were found to be eligible, of which 96 studies reported on overall survival (OS) and 19 studies reported on disease-free survival (DFS) with relation to Ki-67 expression in lung cancer patients.

**Results:**

The pooled hazard ratio (HR) indicated that a high Ki-67 level could be a valuable prognostic factor for lung cancer (HR = 1.122 for OS, *P* < 0.001 and HR = 1.894 for DFS, P < 0.001). Subsequently, the results revealed that a high Ki-67 level was significantly associated with clinical parameters of lung cancer including age (odd ratio, OR = 1.246 for older patients, *P* = 0.018), gender (OR = 1.874 for males, *P* < 0.001) and smoking status (OR = 3.087 for smokers, P < 0.001). Additionally, significant positive correlations were found between Ki-67 overexpression and poorer differentiation (OR = 1.993, *P* = 0.003), larger tumor size (OR = 1.436, P = 0.003), and higher pathologic stages (OR = 1.867 for III-IV, *P* < 0.001). Furthermore, high expression of Ki-67 was found to be a valuable predictive factor for lymph node metastasis positive (OR = 1.653, P < 0.001) and advanced TNM stages (OR = 1.497 for stage III-IV, *P* = 0.024). Finally, no publication bias was detected in any of the analyses.

**Conclusions:**

This study highlights that the high expression of Ki-67 is clinically relevant in terms of the prognostic and clinicopathological characteristics for lung cancer. Nevertheless, more prospective well-designed studies are warranted to validate these findings.

**Electronic supplementary material:**

The online version of this article (10.1186/s12931-018-0843-7) contains supplementary material, which is available to authorized users.

## Background

Lung cancer is the most frequent diagnosed malignant neoplasms, and it was the first cause of cancer death in 2016 globally [1]. In the United States, lung cancer accounted for 27% of all cancer deaths in 2016. Non-small cell lung cancer (NSCLC), accounting for over 80% of all lung cancers, is the major cause of death worldwide [2]. Although the treatment of NSCLC patients includes surgery, radiotherapy and chemotherapy, the progress in lung cancer treatment is still slow, for which the 5-year relative survival is currently 18%. Diagnosis at an advanced stage is the major reason for this low survival rate [3]. Several prognostic factors were well characterized in lung cancer including sex, age, loss of weight, TNM stage, LDH, neutrophilia, haemoglobin as well as serum calcium [4]. Importantly, The IASLC Lung Cancer Staging Project in 2015 also carried out the 8th edition of the anatomic classification of lung cancer, which redefined the tumor-size cut-points in TNM stage for the lung cancer patients. The prognostic value of reclassification of tumor size were confirmed in 70,967 non–small-cell lung cancer patients from 1999 to 2010 [5]. To improve the survival of lung cancer patients, the choice of targeted treatments is increasingly being based on oncogenic drivers including ALK rearrangements, KRAS and epidermal growth factor receptor (EGFR) mutations [6, 7]. Additionally, BRAF mutations represent promising new therapeutic targets for lung cancer [8]. Likewise, several driver biomarkers also shed new light on the target treatment for lung cancer patients such as Her2 [9], NUT [10], DDR2 [11], FGFR1 [12], and PTEN [13]. Furthermore, several new molecular targets been highlighted in lung cancer, including ROS1 fusions [14], NTRK1 fusions [15] and exon 14 skipping mutations [16]. Recently, the checkpoint inhibitors targeting programmed death protein 1 (PD-1) have shown for durable clinical responses in NSCLC patients with advanced stage [17]. The immunomodulatory monoclonal antibodies against cytotoxic T-lymphocyte-associated antigen 4 (CTLA-4) also present the promising results for the treatment of advanced -stages lung cancer patients. However, due to the complex molecular mechanism of lung cancer, the identification of biomarkers in a large proportion of lung cancer patients is still required for a deeper understanding of the underlying epigenetic heterogeneity, and this will benefit the discovery of targeted therapies against lung cancer.

Ki-67, encoded by the MKI67 gene, is expressed throughout the cell cycle in proliferating but absent in in quiescent (G0) cells [18]. Ki-67 appears in the middle or late G1 stage, and then its expression increases through the S and G2 stage until it reaches a peak during the M stage [19]. The high expression of Ki-67 may contribute to aggressive and infiltrative growth of lung squamous carcinoma (SQC), cervical SQC and laryngeal SQC [20–22]. In addition, overexpression of Ki-67 has been positively associated with lymph node metastasis in gastric carcinoma and breast cancer [23, 24]. Ki-67 expression has been reported to be associated with a poor outcome in many malignancies including prostate, bladder and breast cancer [25–29].

Although two meta-analyses have previously reported that Ki-67 could be a possible indicator of short-term survival in lung cancer patients [30, 31], studies on a larger number of lung cancer patients and more reliable evidence are still needed to confirm the prognostic and clinicopathological role of Ki-67 for patients with lung cancer. Thus, our investigation aimed to evaluate the prognostic value and clinicopathological significance of Ki-67 in lung cancer patients via the review of previously published articles.

## Results

### Study selection and characteristics

**Fig. 1:**
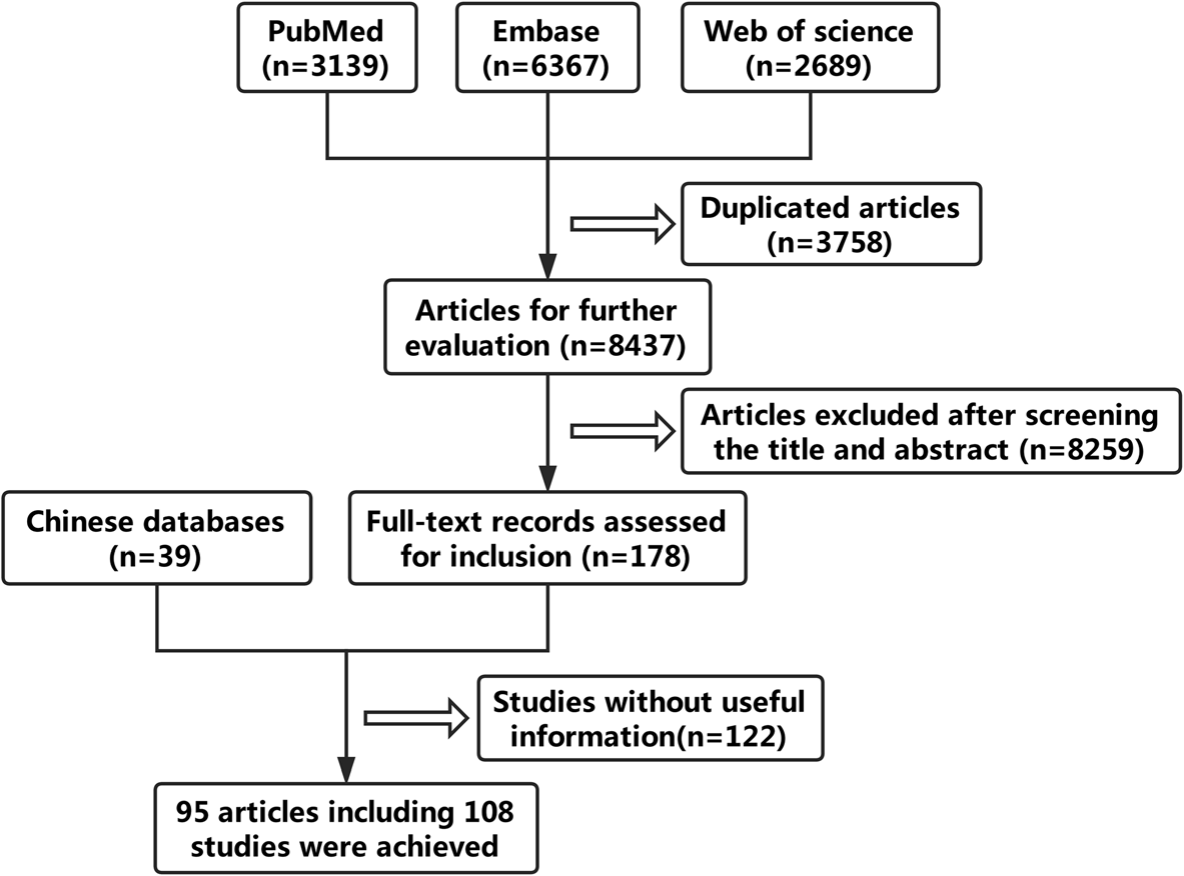
Flow chart of study selection process

As shown in Fig. 1, two investigators read the full text and considered 108 studies in 95 articles [18, 25, 32–124] consisting of 14,732 patients as applicable. The baseline characteristics of the included articles are indicated in Table 1. Ninety-six studies included data regarding OS, and nineteen studies included data regarding DFS. The number of cohorts of each study ranged from 32 to 778. In terms of the study region, 62 studies were from Asia, 30 were from Europe, and 15 were from America. In total, 104 studies consisted of 14,596 NSCLC patients: of these, 28 studies reported on ADC patients, and 10 studies reported on SQC patients. All the studies detected Ki-67 reactivity using IHC.

**Table 1:** Characteristics of studies included into the meta-analysis

Study(author/year)	Region	Tumor stage	Histological type	Patients	Sample size	Cutoff value	Sample type	Assay	NOS score	Extract method	Survival
Scagliotti 1993 [91]	Italy	I-IIIA	NSCLC	111	Large	25%	Tumor tissue	IHC	7	Survival curve	OS
Pence 1993 [120]	USA	I-IV	NSCLC	61	Small	4%	Tumor tissue	IHC	7	Survival curve	OS
Fontanini 1996 [46]	Italy	NA	NSCLC	70	Small	30%	Tumor tissue	IHC	7	Survival curve	OS
Bohm 1996 [37]	Germany	NA	SCLC	32	Small	27%	Tumor tissue	IHC	7	Original data	OS
Harpole 1996 [50]	USA	I	NSCLC	275	Large	7%	Tumor tissue	IHC	9	Multivariate	OS
Pujoll 1996 [88]	France	I-IV	LC	97	Small	NA	Tumor tissue	IHC	7	Survival curve	OS
Mehdi 1999 [119]	USA	I-IV	NSCLC	203	Large	25%	Tumor tissue	IHC	9	Multivariate	OS and DFS
Demarchi 1999 [41]	Brazil	I-III	ADC	64	Small	22%	Tumor tissue	IHC	7	Original data	OS
Dingemans 1999 [42]	Netherland	I-III	SCLC	93	Small	30%	Tumor tissue	IHC	7	Survival curve	OS
Wang 1999 [99]	China	NA	NSCLC	85	Small	30%	Tumor tissue	IHC	7	Survival curve	OS
Shiba 2000 [92]	Japan	I-III	NSCLC	95	Small	20%	Tumor tissue	IHC	7	Univariate	OS
Hommura 2000 [54]	Japan	I-II	SQC	91	Small	30%	Tumor tissue	IHC	9	Multivariate	OS
Hommura 2000 [54]	Japan	I-II	NSCLC	124	Large	30%	Tumor tissue	IHC	9	Multivariate	OS
Nguyen 2000 [80]	Czech Republic	I-IV	NSCLC	89	Small	30%	Tumor tissue	IHC	7	Survival curve	OS
Puglisi 2001 [87]	Italy	I-III	NSCLC	81	Small	30%	Tumor tissue	IHC	9	Multivariate	OS
Hayashi 2001 [52]	Japan	I-IV	NSCLC	98	Small	13%	Tumor tissue	IHC	9	Multivariate	OS
Pelosi 2001 [84]	Italy	I	SQC	119	Large	NA	Tumor tissue	IHC	9	Multivariate	OS and DFS
Ramnath 2001 [121]	USA	I-IV	NSCLC	160	Large	24%	Tumor tissue	IHC	7	Univariate	OS
Ramnath 2001 [121]	USA	I-IV	NSCLC	41	Small	50%	Tumor tissue	IHC	7	Univariate	OS
Wang 2001 [101]	China	I-IV	LC	166	Large	18%	Tumor tissue	IHC	7	Survival curve	OS
Mojtahedzadeh 2002 [78]	Japan	I-III	ADC	141	Large	10%	Tumor tissue	IHC	8	Multivariate	OS
Minami 2002 [76]	Japan	I	ADC	47	Small	20%	Tumor tissue	IHC	8	Multivariate	OS
Takahashi 2002 [94]	Japan	I-IV	NSCLC	62	Small	25%	Tumor tissue	IHC	9	Multivariate	DFS
Wakabayashi 2003 [97]	Japan	I-IV	NSCLC	140	Large	13%	Tumor tissue	IHC	9	Multivariate	OS
Pelosi 2003 [85]	UK	I	ADC	96	Small	NA	Tumor tissue	IHC	9	Multivariate	OS
Haga 2003 [49]	Japan	I	ADC	58	Small	10%	Tumor tissue	IHC	9	Multivariate	OS
Hashimoto 2003 [51]	Japan	I-III	ADC	122	Large	20%	Tumor tissue	IHC	8	Multivariate	OS
Poleri 2003 [86]	Argentina	I	NSCLC	50	Small	67%	Tumor tissue	IHC	7	Survival curve	DFS
Matheus 2004 [75]	Brazil	I-III	ADC	33	Small	22%	Tumor tissue	IHC	7	Original data	OS
Niemiec 2004 [81]	Poland	I-III	SQC	78	Small	28%	Tumor tissue	IHC	7	Survival curve	OS
Ahn 2004 [33]	Korea	II-IIIA	NSCLC	65	Small	15%	Tumor tissue	IHC	9	Multivariate	OS
Huang 2005 [55]	Japan	I	NSCLC	97	Small	25%	Tumor tissue	IHC	8	Multivariate	OS
Gasinska 2005 [48]	Poland	I-III	SQC	81	Small	39%	Tumor tissue	IHC	9	Multivariate	OS
Wang 2005 [100]	China	I-III	NSCLC	51	Small	5%	Tumor tissue	IHC	7	Survival curve	OS
Dong 2005 [43]	Japan	I-IV	ADC	131	Large	18%	Tumor tissue	IHC	9	Multivariate	OS
Niemiec 2005 [81]	Poland	I-III	SQC	78	Large	28%	Tumor tissue	IHC	7	Survival curve	OS
Huang 2005 [55]	Japan	II-III	NSCLC	76	Small	25%	Tumor tissue	IHC	8	Multivariate	OS
Tsubochi 2006 [64]	Japan	I-III	NSCLC	219	Large	20%	Tumor tissue	IHC	9	Multivariate	OS
Yang 2006 [110]	USA	I-III	NSCLC	128	Large	25%	Tumor tissue	IHC	9	Multivariate	OS
Nozawa 2006 [82]	Japan	IV	ADC	35	Small	40%	Tumor tissue	IHC	7	Survival curve	OS
Maddau 2006 [118]	Italy	II-III	NSCLC	88	Large	25%	Tumor tissue	IHC	7	Univariate	OS
Maddau 2006 [118]	Italy	I	NSCLC	92	Large	25%	Tumor tissue	IHC	7	Univariate	OS
Inoue 2007 [58]	Japan	I-III	ADC	97	Small	5%	Tumor tissue	IHC	9	Multivariate	DFS
Mohamed 2007 [77]	Japan	I-IV	NSCLC	61	Small	20%	Tumor tissue	IHC	9	Multivariate	OS
Zhou 2007 [116]	China	I-II	NSCLC	70	Small	NA	Tumor tissue	IHC	6	Multivariate	OS
Morero 2007 [79]	Argentina	III	NSCLC	32	Small	66%	Tumor tissue	IHC	7	Survival curve	OS
Yoo 2007 [112]	Korea	I-III	NSCLC	219	Large	30%	Tumor tissue	IHC	9	Multivariate	OS
Fujioka 2008 [47]	Japan	I	ADC	73	Small	14%	Tumor tissue	IHC	9	Multivariate	OS
Imai 2008 [57]	Japan	I	NSCLC	248	Large	25%	Tumor tissue	IHC	9	Multivariate	OS
Woo 2008 [103]	Japan	Ia	ADC	131	Large	10%	Tumor tissue	IHC	8	Univariate	DFS
Woo 2008 [103]	Japan	Ib	ADC	59	Small	10%	Tumor tissue	IHC	8	Univariate	DFS
Saad 2008 [89]	USA	I-III	NSCLC	54	Small	30%	Tumor tissue	IHC	7	Survival curve	OS
Kaira 2008 [124]	Japan	I-III	NSCLC	321	Large	25%	Tumor tissue	IHC	9	Multivariate	OS
Ikeda 2008 [56]	Japan	I-III	NSCLC	200	Large	5%	Tumor tissue	IHC	7	Univariate	OS and DFS
Anami 2009 [34]	Japan	I-IV	ADC	139	Large	10%	Tumor tissue	IHC	8	Multivariate	OS
Yuan 2009 [113]	China	I-III	NSCLC	140	Large	25%	Tumor tissue	IHC	8	Multivariate	OS
Kaira 2009 [62]	Japan	1	ADC	139	Large	20%	Tumor tissue	IHC	9	Multivariate	OS
Erler 2010 [44]	USA	NA	SCLC	68	Small	50%	Tumor tissue	IHC	7	Survival curve	OS
Filipits 2011 [45]	Austria	I-III	NSCLC	778	Large	NA	Tumor tissue	IHC	9	Multivariate	OS and DFS
Werynska 2011 [102]	Poland	I-IV	NSCLC	145	Large	25%	Tumor tissue	IHC	7	Univariate	OS
Yamashita 2011 [109]	Japan	I	NSCLC	44	Small	5%	Tumor tissue	IHC	8	Multivariate	DFS
Wu 2011 [106]	China	I-IV	NSCLC	160	Large	10%	Tumor tissue	IHC	8	Multivariate	OS
Oka 2011 [83]	Japan	I-III	ADC	183	Large	20%	Tumor tissue	IHC	9	Multivariate	DFS
Sterlacci 2011 [122]	Austria	I-IV	NSCLC	386	Large	3%	Tumor tissue	IHC	9	Multivariate	OS
Werynska 2011 [102]	Poland	NA	NSCLC	145	Large	25%	Tumor tissue	IHC	7	Univariate	OS
Liu 2012 [71]	China	I-IV	NSCLC	494	Large	50%	Tumor tissue	IHC	9	Multivariate	OS
Wang 2012 [98]	China	NA	SCLC	42	Small	10%	Tumor tissue	IHC	7	Survival curve	OS
Liu 2012 [71]	China	I-IV	ADC	97	Small	10%	Tumor tissue	IHC	7	Survival curve	OS
Wu 2012 [105]	China	I-IV	ADC	309	Large	50%	Tumor tissue	IHC	9	Multivariate	OS
Salvi 2012 [90]	Italy	I-III	NSCLC	81	Small	15%	Tumor tissue	IHC	9	Multivariate	OS
Yang 2012 [111]	China	I-III	NSCLC	68	Small	38%	Tumor tissue	IHC	7	Survival curve	OS
Wu 2013 [104]	China	I-IV	NSCLC	192	Large	10%	Tumor tissue	IHC	9	Multivariate	OS and DFS
Maki 2013 [74]	Japan	I	ADC	105	Large	15%	Tumor tissue	IHC	9	Multivariate	DFS
Lei 2013 [69]	China	I-IV	NSCLC	279	Large	30%	Tumor tissue	IHC	9	Multivariate	OS
Berghoff 2013 [36]	Austria	I-IV	NSCLC	230	Large	40%	Tumor tissue	IHC	9	Multivariate	OS
Ji 2013 [61]	China	I-III	NSCLC	67	Small	5%	Tumor tissue	IHC	9	Multivariate	OS
Kobyakov 2013 [68]	USA	I-III	SQC	118	Large	30%	Tumor tissue	IHC	7	Survival curve	OS
Zu 2013 [117]	China	I-III	ADC	96	Small	25%	Tumor tissue	IHC	7	Survival curve	OS
Liu 2013 [70]	China	I-III	NSCLC	105	Large	50%	Tumor tissue	IHC	8	Multivariate	OS
Hokka 2013 [53]	Japan	I-IV	ADC	125	Large	NA	Tumor tissue	IHC	9	Multivariate	OS
Xue 2013 [73]	China	I-III	NSCLC	83	Small	50%	Tumor tissue	IHC	8	Multivariate	OS
Zhong 2014 [115]	China	I-IV	NSCLC	270	Large	50%	Tumor tissue	IHC	8	Multivariate	OS
Ahn 2014 [32]	Korea	I-III	NSCLC	108	Large	40%	Tumor tissue	IHC	9	Multivariate	DFS
Shimizu 2014 [93]	Japan	I-III	SQC	32	Small	10%	Tumor tissue	IHC	8	Multivariate	DFS
Shimizu 2014 [93]	Japan	I-III	ADC	52	Small	10%	Tumor tissue	IHC	8	Multivariate	DFS
Kim 2014 [136]	Korea	I-IV	ADC	122	Large	10%	Tumor tissue	IHC	7	Univariate	OS
Tsoukalas 2014 [95]	Greece	I-IV	NSCLC	112	Large	NA	Tumor tissue	IHC	9	Multivariate	OS
Kawatsu 2014 [63]	Japan	I-IV	NSCLC	183	Large	10%	Tumor tissue	IHC	8	Multivariate	OS
Corzani 2014 [39]	Italy	III	NSCLC	50	Small	50%	Tumor tissue	IHC	8	Multivariate	OS
Warth 2014 [18]	Germany	I-IV	ADC	482	Large	25%	Tumor tissue	IHC	7	Univariate	OS and DFS
Warth 2014 [18]	Germany	I-IV	SQC	233	Large	50%	Tumor tissue	IHC	8	Multivariate	OS
Tabata 2014 [25]	Japan	I-IV	NSCLC	74	Small	10%	Tumor tissue	IHC	9	Multivariate	OS
Xu 2014 [108]	China	I-IV	ADC	80	Small	5%	Tumor tissue	IHC	7	Survival curve	OS
Liu 2014 [70]	China	I-IV	NSCLC	96	Small	30%	Tumor tissue	IHC	7	Survival curve	OS
Ji 2014 [60]	China	I-III	NSCLC	83	Small	NA	Tumor tissue	IHC	8	Multivariate	OS
Shimizu 2014 [93]	Japan	I-IV	SQC	32	Large	10%%	Tumor tissue	IHC	9	Multivariate	OS
Shimizu 2014 [93]	Japan	I-IV	ADC	52	Large	10%%	Tumor tissue	IHC	9	Multivariate	OS
Kobierzycki 2014 [67]	Poland	I-IV	NSCLC	218	Large	25%	Tumor tissue	IHC	6	Univariate	OS
Xu 2014 [107]	China	I-III	NSCLC	114	Large	50%	Tumor tissue	IHC	7	Univariate	OS
Zhang 2015 [114]	China	I-IV	ADC	616	Large	NA	Tumor tissue	IHC	8	Multivariate	OS
Gobbo 2015 [40]	Italy	NA	NSCLC	383	Large	20%	Tissue microarray	IHC	7	Univariate	OS
Stewart 2015 [123]	USA	II-IIIA	NSCLC	230	Large	NA	Tumor tissue	IHC	7	Univariate	DFS
Vigouroux 2015 [96]	France	I-IV	NSCLC	190	Large	40%	Tumor tissue	IHC	7	Survival curve	OS
Jethon 2015 [59]	Poland	I-IV	SQC	89	Small	25%	Tumor tissue	IHC	7	Univariate	OS
Jethon 2015 [59]	Poland	I-IV	ADC	98	Small	25%	Tumor tissue	IHC	7	Univariate	OS
Apostolova 2016 [35]	Germany	I-IV	NSCLC	83	Small	75%	Tumor tissue	IHC	8	Multivariate	OS
Cardona 2016 [38]	USA	NA	NSCLC	144	Large	30%	Tumor tissue	IHC	7	Original data	OS

### Prognostic value of Ki-67 for survival outcome in lung cancer patients

**Fig. 2:**
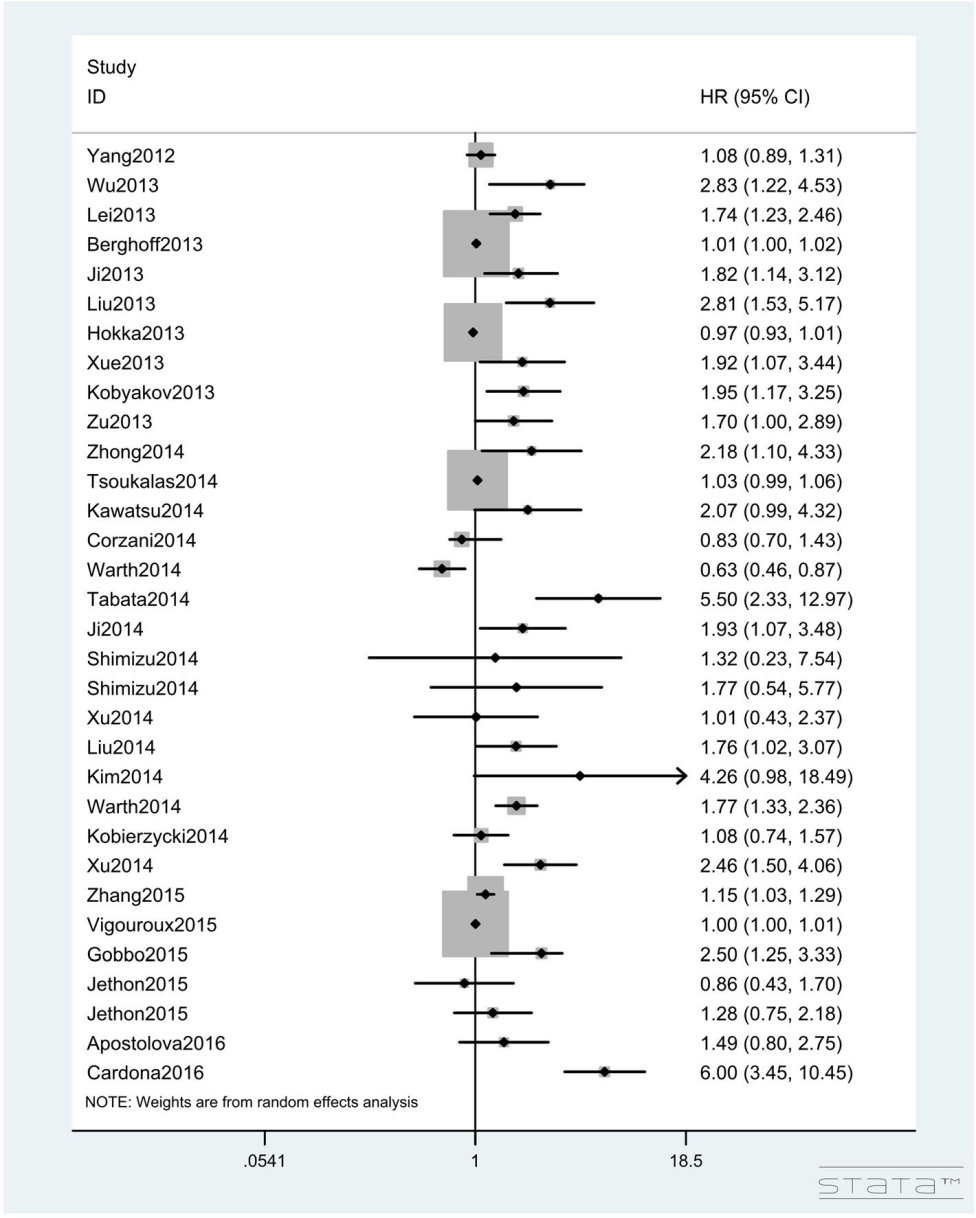
Hazard ratios and 95% CIs of studies included in meta-analysis of OS

**Fig. 3:**
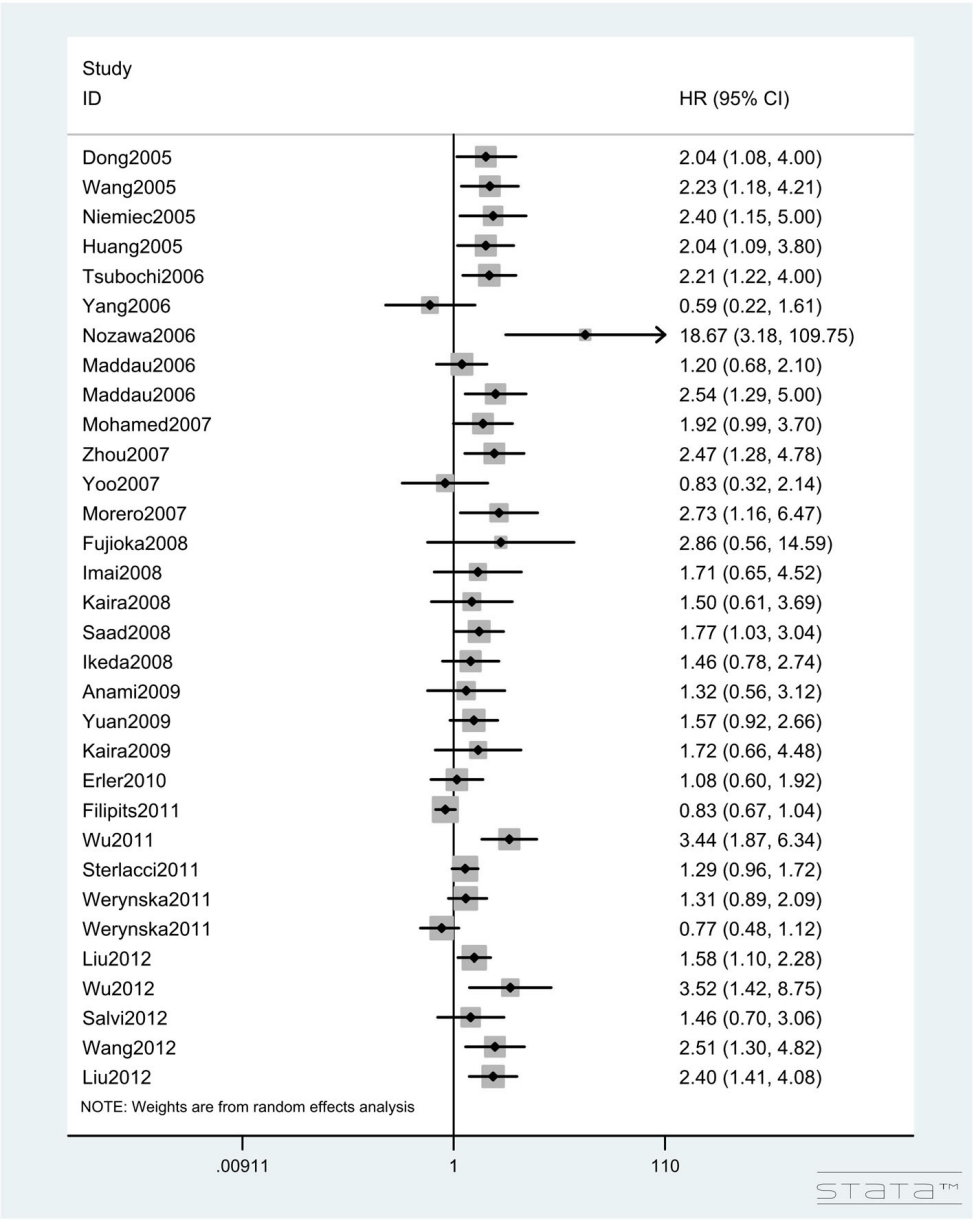
Hazard ratios and 95% CIs of studies included in meta-analysis of OS

**Fig. 4:**
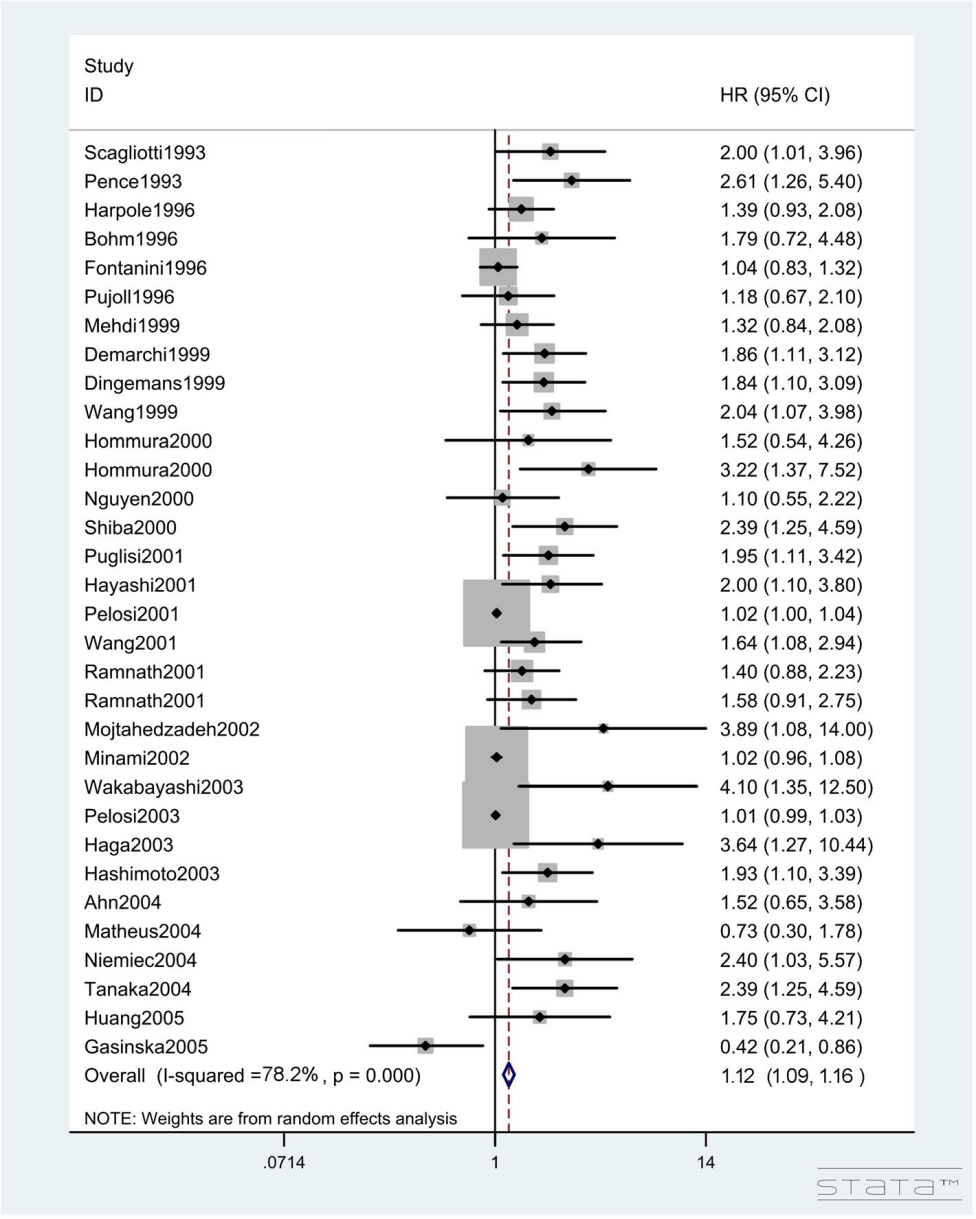
Hazard ratios and 95% CIs of studies included in meta-analysis of OS

**Fig. 5:**
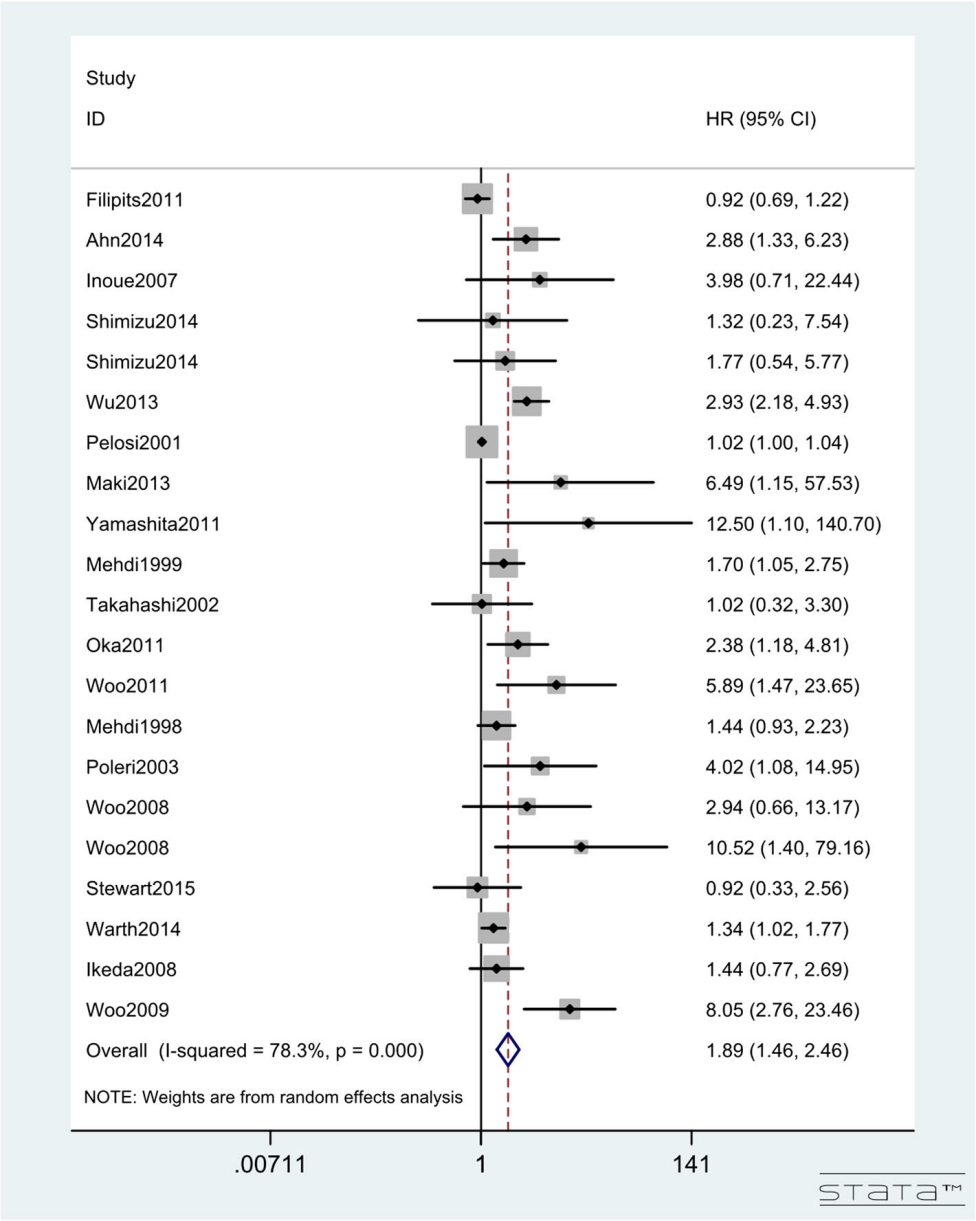
Hazard ratios and 95% CIs of studies included in meta-analysis of DFS

In total, 95 studies with 13,678 lung cancer patients investigated the impact of Ki-67 expression on OS (Table 2). The pooled HR of the total population for OS was 1.122 (95%CIs: 1.089–1.156, Z = 7.56, *P* < 0.001; I^2^ = 78.20%, P < 0.001, Figs. 2, 3 and 4), showing that a high Ki-67 level indicates worse outcome for lung cancer patients. Furthermore, the correlation of high Ki-67 expression with DFS in 3127 lung cancer patients was then analyzed (Table 3). For the total study population, worse DFS (HR = 1.894, 95%CIs: 1.456–2.463, Z = 4.76, P < 0.001, Fig. 5) was observed among patients with high expression of Ki-67, while the heterogeneity using the random effects model was obvious (I^2^ = 78.30%, P < 0.001). To investigate the source of heterogeneity, subgroup analyses of publication year, region, histological type, sample size, cut-off value of Ki-67 and estimated method for HR determination were performed. From the subgroup analysis of OS, no heterogeneity was found in the small cell lung cancer (SCLC) group (I^2^ = 22.30%, *P* = 0.277). Next, a reduction in heterogeneity was observed after performing subgroup analysis of DFS according to the study region, especially in the studies from America (I^2^ = 8.40%, *P* = 0.351) and Asia (I^2^ = 25.60%, *P* = 0.179). As indicated in the subgroup of cutoff value, there was a low degree of heterogeneity in both Ki-67 low expression (I^2^ = 25.30%, *P* = 0.196) and high expression groups (I^2^ = 19.40%, *P* = 0.287). Furthermore, we also performed meta-regression analysis to explore the original of the heterogeneity in the studies. Consistent with the subgroup analysis, the results revealed that the regions and cut-off values might be the potential bias for the heterogeneity (*P* = 0.017 and *P* = 0.022, respectively). Altogether, we concluded that the different regions and inconsistent cut-off values might have contributed to the heterogeneity in the results of analyses for DFS. The regression also revealed that the heterogeneity originated from regions and inconsistent cut-off values (Table 4).

**Table 2:** Summarized HRs of overall and subgroup analyses for OS

Stratified analysis	Study(N)	HR	z	P	Heterogeneity		
					I^2^	P	Estimated method
OS	95	1.122(1.089–1.156)	7.56	< 0.001	78.20%	< 0.001	Random-effect
Subgroup analysis							
Publication year							
Early year(~ 2007)	44	1.307(1.212–1.408)	7.01	< 0.001	72.50%	< 0.001	Random-effect
Later year(2007~ 2016)	51	1.101(1.063–1.142)	5.28	< 0.001	81.20%	< 0.001	Random-effect
Region							
Europe	30	1.021(1.001–1.042)	2.05	0.041	71.30%	< 0.001	Random-effect
America	13	1.671(1.266–2.205)	3.63	< 0.001	66.60%	< 0.001	Random-effect
Asia	52	1.821(1.623–2.043)	10.21	< 0.001	78.20%	< 0.001	Random-effect
Histological type							
SCLC	4	1.023(1.004–1.042)	3.28	0.001	22.30%	0.277	Fixed-effect
NSCLC	89	1.113(1.081–1.147)	7.11	< 0.001	78.70%	< 0.001	Random-effect
ADC	22	1.219(1.113–1.336)	4.26	< 0.001	76.20%	< 0.001	Random-effect
SQC	9	1.115(0.806–1.542)	0.66	0.512	74.40%	< 0.001	Random-effect
Sample size							
< 100	45	1.486(1.340–1.649)	7.71	< 0.001	73.10%	< 0.001	Random-effect
> 100	50	1.083(1.049–1.119)	4.90	< 0.001	81.50%	< 0.001	Random-effect
Cutoff value							
L(< 20%)	34	1.962(1.622–2.373)	6.95	< 0.001	74.50%	< 0.001	Random-effect
H(≥20%)	52	1.144(1.094–1.197)	5.91	< 0.001	78.90%	< 0.001	Random-effect
Estimated method							
Original data	4	2.043(0.868–4.808)	1.64	0.102	83.90%	< 0.001	Random-effect
Survival curve	23	1.629(1.368–1.940)	5.47	< 0.001	76.30%	< 0.001	Random-effect
HR(univariate)	16	1.511(1.236–1.847)	4.02	< 0.001	56.10%	0.004	Random-effect
HR(multivariate)	53	1.108(1.063–1.155)	4.87	< 0.001	75.30%	< 0.001	Random-effect

**Table 3:** Summarized HRs of overall and subgroup analyses for DFS

Stratified analysis	Study(N)	HR	Z	P	Heterogeneity		
					I^2^	P	Estimated method
DFS	21	1.894(1.456–2.463)	4.76	< 0.001	78.30%	< 0.001	Random-effect
Subgroup analysis for DFS							
Publication year							
Early year(~ 2007)	6	1.428(0.992–2.055)	1.92	0.055	62.10%	0.022	Random-effect
Later year(2007~ 2016)	15	2.237(1.54–3.249)	4.23	< 0.001	72.00%	< 0.001	Random-effect
Region							
Europe	3	1.023(1.005–1.041)	2.51	0.012	53.40%	0.117	Fixed-effect
America	4	1.559(1.155–2.105)	2.9	0.004	8.40%	0.351	Fixed-effect
Asia	14	2.673(2.096–3.409)	7.92	< 0.001	25.60%	0.179	Fixed-effect
Histological type							
SCLC							
NSCLC	21	1.894(1.456–2.463)	4.76	< 0.001	78.30%	< 0.001	Random-effect
ADC	9	3.186(1.797–5.650)	3.96	< 0.001	62.10%	0.007	Random-effect
SQC	2	1.022(1.004–1.04)	2.42	0.015	0.00%	0.774	Random-effect
Sample size							
< 100	7	2.455(1.392–4.330)	3.10	< 0.001	20.30%	0.28	Fixed-effect
> 100	14	1.770(1.340–2.338)	4.02	< 0.001	82.80%	< 0.001	Random-effect
Cutoff value							
L(< 20%)	12	2.783(2.141–3.619)	7.64	< 0.001	25.30%	0.196	Fixed-effect
H(≥20%)	6	1.514(1.243–1.844)	4.12	< 0.001	19.40%	0.287	Fixed-effect
Estimated method							
Survival curve	2	1.595(1.053–2.416)	2.21	0.027	52.60%	0.146	Fixed-effect
HR(univariate)	6	2.126(1.156–3.909)	2.43	0.015	67.40%	0.009	Random-effect
HR(multivariate)	13	1.892(1.328–2.698	3.53	< 0.001	79.90%	< 0.001	Random-effect

**Table 4:** Meta-regression for the OS and DFS analysis

Variables	HR	Standard Error	t	P > |t|	Lower limit	Upper limit
OS						
Year	0.999	0.104	−0.010	0.991	0.811	1.230
Region	0.835	0.061	−2.450	0.017	0.722	0.967
Cancer type	0.954	0.045	−0.990	0.326	0.868	1.049
Sample size	1.108	0.122	0.930	0.353	0.890	1.380
Cutoff value	0.777	0.084	−2.330	0.022	0.626	0.964
Statistical method	1.045	0.044	1.050	0.295	0.961	1.137
DFS						
Year	2.011	1.525	0.920	0.377	0.379	10.678
Region	0.591	0.289	−1.070	0.306	0.201	1.736
Cancer type	1.125	0.401	0.330	0.747	0.513	2.467
Sample size	0.793	0.348	−0.530	0.607	0.302	2.081
Cutoff value	0.767	0.363	−0.560	0.587	0.271	2.172
Statistical method	0.994	0.221	−0.030	0.979	0.609	1.622

### The correlation of Ki-67 expression and clinicopathological features in lung cancer patients

An association of Ki-67 expression with age in 2506 lung cancer patients was identified using the fixed effects model in 19 studies, and higher Ki-67 expression was found to be more common in older patients (OR = 1.246, 95%CIs: 1.039–1.494; Z = 2.37, *P* = 0.018, I^2^ = 0.00%, *P* = 0.967, Table 5 and Additional file 1: Figure S1). Subsequently, the results revealed significant differences in Ki-67 level between male and female (OR = 1.874, 95%CIs: 1.385–2.535; Z = 4.07, *P* < 0.001, I^2^ = 69.70%, *P* < 0.0001, Additional file 1: Figure S1B). Meta-analysis of 15 studies including 2152 lung cancer patients revealed a positive association between high Ki-67 level and smoking history (OR = 3.087, 95%CIs: 2.504–3.806, Z = 10.56, P < 0.001; I2 = 39.40%, *P* = 0.064, Additional file 1: Figure S1C). According to the histological type, a pooled OR of 0.397 (95%CIs: 0.236–0.667) indicated that Ki-67 expression was significantly higher in ADC compared with that in SQC (Z = 3.49, *P* < 0.001; I^2^ = 81.20%, P < 0.001, Additional file 2: Figure S2A). Next, tumor differentiation was considered. The results from 11 studies enrolling 1731 lung cancer patients showed that an elevated Ki-67 level was associated with poor differentiation, with a pooled OR of 1.993 (95%CIs:1.262–3.146, Z = 2.96, *P* = 0.003; I^2^ = 66.30%, *P* = 0.001, Additional file 2: Figure S2B). A total of 13 studies with 1851 individuals were analyzed in this meta-analysis, and the results showed that a higher Ki-67 level was positively associated with the pathologic stage III/IV with a low degree of heterogeneity (OR = 1.867, 95%CIs: 1.498–2.327, Z = 5.56, P < 0.001; I^2^ = 23.1%, *P* = 0.210, Additional file 2: Figure S2C). A trend toward positive correlation was found between a high Ki-67 level and larger tumor size in 12 studies based on 1707 lung cancer patients, with a pooled OR of 1.436 (95%CIs:1.127~ 1.290,, Z = 2.93, P = 0.003; I^2^ = 0.00%, *P* = 0.876, Additional file 3: Figure S3A). Twenty-three studies comprising 2994 cases were used for meta-analysis of Ki-67 expression and lymph node metastasis, and the pooled OR indicated that a high Ki-67 level was significantly correlated with lymph node metastasis positive (OR = 1.653, 95%CIs: 1.285–2.127, Z = 3.91, *P* < 0.0001; I^2^ = 46.70%, *P* = 0.008, Additional file 3: Figure S3B). The association of Ki-67 expression and TNM stage was then incorporated into the meta-analysis. Eight studies with 736 patients showed a trend for correlation between Ki-67 overexpression and advanced TNM stages, with a pooled OR of 1.50 (95%CIs:1.053~ 2.126, Z = 2.25, *P* = 0.024; I^2^ = 36.90%, *P* = 0.134, Additional file 3: Figure S3C). In the meta-analysis, no association between Ki-67 and tumor stage was observed in lung cancer patients (OR = 1.287, 95%CIs:0.882–1.877, Z = 1.31, *P* = 0.191; I^2^ = 55.30%, *P* = 0.013). Additionally, analysis of four selected studies using the random effects model did not reveal any significance for the association between Ki-67 expression and metastasis (OR = 2.609, 95%CIs: 0.667–10.204, Z = 1.38, *P* = 0.168) or invasion (OR = 0.993, 95%CIs: 0.511–1.930, Z = 0.02, *P* = 0.984; I^2^ = 14.20%, *P* = 0.312).

**Table 5:** Main results for meta-analysis between Ki-67 and clinicopathological features in lung cancer

Clinicopathological features	Study(n)	Pooled OR(95%CIs)	z	P	Heterogeneity			Publication bias
					I^2^	P	Estimated method	P
Age	19	1.246(1.039–1.494)	2.37	0.018	0.00%	0.967	Fixed-effect	0.234
Gender	26	1.874(1.385–2.535)	4.07	< 0.001	69.70%	0.000	Random-effect	1.000
Histological type	16	0.397(0.236–0.667)	3.49	< 0.001	81.20%	0.000	Random-effect	0.324
Differentiation	11	1.993(1.262–3.146)	2.96	0.003	66.30%	0.001	Random-effect	0.893
Pathologic stage	13	1.867(1.498–2.327)	5.56	< 0.001	23.10%	0.210	Fixed-effect	1.000
Tumor size	12	1.436(1.127–1.29)	2.93	0.003	0.00%	0.876	Fixed-effect	0.276
Tumor stage	11	1.287(0.882–1.877)	1.31	0.191	55.30%	0.013	Random-effect	0.086
Metastasis	4	2.609(0.667–10.204)	1.38	0.168	64.50%	0.038	Random-effect	0.428
Lymph node	23	1.653(1.285–2.127)	3.91	< 0.001	46.70%	0.008	Random-effect	0.876
TNM stage	8	1.497(1.053–2.126)	2.25	0.024	36.90%	0.134	Fixed-effect	0.187
Invasion	3	0.993(0.511–1.930)	0.02	0.984	14.20%	0.312	Fixed-effect	0.308
Smoking	15	3.087(2.504–3.8060)	10.56	< 0.001	39.40%	0.064	Fixed-effect	0.711

### Publication bias

**Fig. 6:**
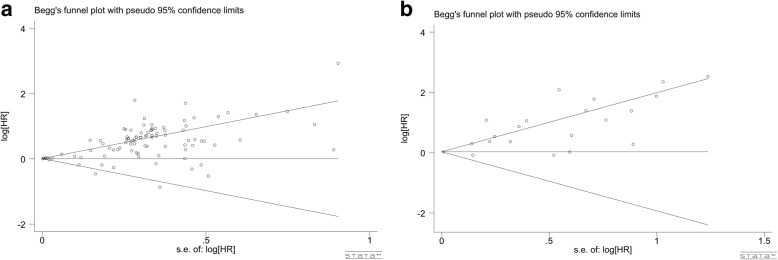
Funnel plots for publication bias of OS and DFS meta-analysis

To identify potential publication bias, Begg’s test and funnel plots were used. No publication bias was found in the analysis for OS (*p* = 0.444, Fig. 6a) and DFS (*P* = 0.246, Fig. 6b). Moreover, there was no publication bias among any of the analyses used to correlate Ki-67 expression and clinicopathological characteristics (all *P* > 0.05, Table 5, Additional file 4: Figure S4, Additional file 5: Figure S5, Additional file 6: Figure S6 and Additional file 7: Figure S7).

## Discussion

As previously mentioned, there are two meta-analyses showing that high expression of Ki-67 predicts worse prognosis in lung cancer patients [30] and early-stage NSCLCs [31]. Nevertheless, there is no consensus regarding the clinicopathological significance of Ki-67 in lung cancer patients. Martin et al. performed a meta-analysis on 37 studies to evaluate the prognostic value of Ki-67 in 3983 lung cancer patients in 2004 [30]. Lacking sufficient information for other subtypes of lung cancer and Asian patients, the results from the abovementioned meta-analysis were not convincing. Our meta-analysis includes 108 studies with 14,831 lung cancer patients comprising NSCLC and SCLC cases and thus provides more reliable evidence. Additionally, we also restricted the number of patients in each study to greater than 30 to exclude low-quality studies. To strengthen the evidence, we estimated not only OS data but also DFS to determine the prognostic role of Ki-67 in lung cancer patients. Moreover, multivariate analyses of OS and DFS were performed, and the HR of OS was 1.108(95%CIs: 1.063–1.155), and that for DFS was 1.892(95%CIs: 1.328–2.698), indicating that Ki-67 is an independent prognostic marker for lung cancer. Compared to the previous meta-analysis, our meta-analysis included results on all subtypes of lung cancer and represented broader ethnicity; in addition, subgroups were classified according to region, cut-off value, number of patients and histological type. With the inclusion of high-quality studies and a larger number of patients, the results derived from our study are more convincing.

Ki-67 is present in the active phases of the cell cycle (G1, S, and G2), as well as during mitosis, but it is not expressed in the G0 phase. Thus, it has become an excellent operational marker for the estimation of the proportion of proliferative cells in a given cell population [125]. Our study demonstrated that Ki-67 expression was lower in ADC compared with that in SQC, suggesting it is a useful biomarker for distinguishing ADC from SQC. Consisted with our result, Ki-67 was also revealed to be higher in ADC than in SQC in the same stage, duo to the different tumor biology of histological subtypes in NSCLC [49, 64]. The high-grade Ki-67 was proved to be significantly correlated with a more aggressive tumor infiltration patterns in lung SQC, Indicating the strong association between tumor invasiveness and cell proliferation [20]. It has been reported in the literature that inverted papillomas with high levels of Ki-67 also include squamous cell carcinomas. Regarding the SCLC, Vasudha Murlidhar et al. recently carried out a result that Ki-67 could contribute to the early detection of metastasis in circulating lung cancer cells. Ki-67 was also demonstrated as a potential diagnostic factor for histopathological definition of SCLC [126]. Contrary to our study, previous study revealed that maternal cigarette smoking could dramatically decrease the expression of Ki-67 in cytotrophoblasts [127]. Interestingly, further study also found that Ki-67 was lower expressed in smokers and smokers with COPD compared to the non-smokers. The authors hypothesized that the permanent cellular damage might play a crucial role in the destruction of bronchiolar tissue [128]. Nevertheless, the mechanisms that govern how Ki-67 expression contribute to the tumorigenesis and progression of lung cancer remain to be unveiled.

The authors suspected that Ki-67 might affect cyclin-dependent kinase1 (CDK1), leading to the entry of inverted papilloma cells into the active phase of cell cycle(G1)and resulting in malignant transformation [129]. Interestingly, our study found that Ki-67 expression in male patients was significantly higher comparing with that in female patients. Previous reports have suggested that testosterone can promote the growth of cancer cells that express androgen receptors, which negatively regulates the Ki-67 level in lung cancer patients [130, 131]. Many previous studies have also revealed that Ki-67 is significantly associated with histopathologic parameters in other tumor because of the correlation between proliferation and those parameters [132–134]. It was found that p53 regulated the p53- and Sp1-dependent pathways, leading to the inhibition of Ki-67 promoter [135, 136]. A recent study confirmed that there was a correlation between the specific Ki-67 splice variants and the progression through the cell cycle in cancer cells. Ki-67 might be involved in a putative extranuclear elimination pathway transported to the Golgi apparatus [137]. Based on these results, we concluded that Ki-67 serves as a valuable indicator for the aggressiveness and prognosis of lung cancer.

Heterogeneity was significant in this meta-analysis. To eliminate the heterogeneity, subgroup analyses according to region, cut-off value, number of patients and histological type were carried out using random effects models. As a result, we revealed that the source of heterogeneity originated from the publication region and the cutoff value by the meta-regression analysis. Our meta-analysis was limited to publications in English or Chinese; nevertheless, the researchers typically tend to publish studies with negative results in local journals and in the native language of the study region. In addition, although detailed exclusion criteria were established to avoid duplication, our meta-analysis was not able to avoid the same patient cohorts in different publications. Other methodological factors might also affect heterogeneity, such as the antibody and cut-off value used in the study. Although anti-MIB-1 antibody is the most frequently used antibody in studies, most of the included studies stratified high and low levels of Ki-67 using a median value varying from 3 to 75%, which might have influenced the results. Several studies used the Ki-67 cut-off less than 10% to assess the prognostic impact of Ki-67 after surgical resection with curative intent in early-stages lung cancer patients. Most of the included studies used the median value of Ki-67 index as cutoff value, which could divide the patient into equally the group but did not reflect the clinical relevant use. A cut-off value of Ki-67 maximizing the hazard ratio across the groups could be used for the clinical management for the diagnosis and prognosis of lung cancers. More importantly, Multiple clinical laboratories have reported the Ki-67 cutoff values between 10 and 14% could be recommended as the gold standard identify the high risk of the survival outcome in cancers [138–141]. Additionally, microarrays were used in several studies, for which the sensitivity of assessment of Ki-67 expression is generally poor. Another possibility of bias may be related to the method of extrapolating the HR; the HR extracted from survival curves was less reliable than direct analysis of variance. Additionally, the subgroup analysis for different stages because most of the included studies recruited the lung cancer patients within three or more tumor stages. Thus, we could not classify the patients into the early stages and advanced stages for the subgroup analysis.

## Conclusion

In conclusion, our meta-analysis demonstrated that high expression of Ki-67 is associated with worse prognosis and disease progression in lung cancer patients. Ki-67 can be an independent biological marker for predicting the prognosis of lung cancer patients. Subsequent studies are required to investigate the prognoses and clinical characteristics of lung cancer patients to confirm our findings.

## Materials and methods

### Literature search and selection

The databases that we searched included PubMed, Web of Science, EMBASE, and Chinese datasets (WanFang, China National Knowledge Infrastructure and Chinese VIP) until June 1, 2017. The key words identifying the articles were as follows: (Ki-67 OR Ki67 OR MIB-1 OR “proliferative index” OR “proliferative activity” OR “mitotic index” OR “labeling index” OR “mitotic count” OR “proliferative marker” OR “mitotic figure” OR “mitotic activity”) AND (Cancer OR carcinoma OR adenocarcinoma OR tumour OR tumor OR malignanc* OR neoplas*) AND (Lung OR pulmonary OR respiratory OR respiration OR aspiration OR bronchi OR bronchioles OR alveoli OR pneumocytes OR “air way”).

### Selection criteria

Publications were included if they met the following inclusion criteria: (1) the patients enrolled had been diagnosed with lung cancer; (2) the results for the study included the correlation between Ki-67 and overall survival (OS) or disease-free survival (DFS); (3) the samples used in the studies were human lung tissue, serum or sputum but not animals or cell lines; (4) the techniques used to measure the expression level of Ki-67 in cancer tissue or tumors of the patients were immunohistochemistry (IHC), PCR/RT-PCR, ELISA or western blotting; (5) the study provided hazard ratios (HRs) and their 95% confidence intervals (CIs) or sufficient information for estimating these parameters; (6) the article was fully written in English or Chinese; and (7) the sample size was larger than 30. Studies were excluded if they met the following exclusion criteria:(1) if they included animal experiments or cell lines or were pre-clinical studies, meta-analyses, reviews, comments, conference abstracts, letters or case reports; (2) articles in languages other than English or Chinese; and (3) studies did not include the key information for survival analyses such as HRs and 95%CIs. To avoid data duplication, when the same patient cohort was reported in different publications or the same article was found in different journals, only the most recent and complete publication was included.

### Data extraction and quality assessment

All the articles were independently reviewed and selected by two investigators. Discrepancies were resolved by discussion and arbitrated by a third investigator. The following information was extracted from each publication: first author’s name, year of publication year, pathology type, tumor stage, number of patients, sample type, cut-off value of Ki-67, determination assay, method to extract HR and survival type. Additionally, we also obtained the clinicopathological characteristics of the lung cancer patients in the included studies including age (old/young), gender (male/female), histological type (adenocarcinoma/squamous carcinoma, ADC/SQC), smoking status (smoker/non-smoker), differentiation (poor/well or moderate), pathologic stage (III-IV/I-II), tumor size (large/small), tumor stage (T3–4/T1-T2), metastasis (yes/no), lymph node (N1-Nx/N0), TNM stage (T3–4/T1-T2) and invasion (yes/no). Especially, the tumor stage was used to describe the size and extent of tumor. And the TNM stage were used to define the progression of cancer based on the size and extension tumor, lymphatic involvement and metastasis status. Based on the Newcastle Ottawa Scale (NOS) criteria [142], studies with NOS scores higher than 6 are considered high-quality studies, whereas those with NOS scores less than 5 are defined as low-quality studies. This study was strictly performed according to the Preferred Reporting Items for Systematic Reviews and Meta-Analyses (PRISMA) [143] and the PRISMA checklist were also provided in the Additional file 8.

### Statistical methods

HR and 95%CIs were used to measure the relationship between Ki-67 expression and prognosis of lung cancer patients. The most accurate determination was made when the study directly provided the HRs and 95%CIs. The multivariate HRs were calculated by using the Cox proportional hazards model, which could independently predict the survival outcome for the lung cancer patients. We preferentially chose the multivariate values when the study provided both univariate and multivariate HRs. If the above data were not available, we used the Engauge Digitizer version 4.1 to extract the survival rates from KM curves and estimated the HR according the method as Tierneyet al. described [144, 145]. Moreover, we calculated the HR from the original survival data that the study provided using SPSS. An observed HR > 1 indicated worse prognosis for the lung cancer patients with high expression of Ki-67, if the 95%CIs for the overall HR was not 1, we considered the prognostic effect of Ki-67 on to be statistically significant. Odds ratio (OR) with 95%CIs were used to analyze the degree of association between Ki-67 level and clinicopathological characteristics. Heterogeneity was determined using the χ^2^ and inconsistency (I^2^) tests [146]. I^2^ > 50% or *P* < 0.1 indicated substantial heterogeneity among the studies, in which case a random effects model was applied; otherwise, we utilized the fixed effects model. Then, subgroup analysis and meta-regression analysis were used to investigate any source of heterogeneity. Moreover, publication bias was assessed using Begg’s test and funnel plots, and *P*-values < 0.05 indicated statistically significant publication bias [147].

## Additional files

Additional file 1: **Figure S1.** Forest plots for the relationships between Ki-67 expression and clinicopathological features of patients with lung cancer. A. Age B. Gender C. Histological type. (TIF 4444 kb)

Additional file 2: **Figure S2.** Forest plots for the relationships between Ki-67 expression and clinicopathological features of patients with lung cancer. A. Differentiation B. Pathologic stage C. Tumor size. (TIF 3759 kb)

Additional file 3: **Figure S3.** Forest plots for the relationships between Ki-67 expression and clinicopathological features of patients with lung cancer. A. Lymph node B. TNM stage C. Smoking. (TIF 3186 kb)

Additional file 4: **Figure S4.** Funnel plots for publication bias of clinicopathological features meta-analysis (A~C). A. Age B. Gender C. Histological type. (TIF 20061 kb)

Additional file 5: **Figure S5.** Funnel plots for publication bias of clinicopathological features meta-analysis (D~F). A. Differentiation B. Pathologic stage C. Tumor size. (TIF 21452 kb)

Additional file 6: **Figure S6.** Funnel plots for publication bias of clinicopathological features meta-analysis (G~J). A. Tumor stage B. Lymph node C. TNM stage. (TIF 21299 kb)

Additional file 7: **Figure S7.** Funnel plot for publication bias of clinicopathological features meta-analysis (Smoking status). (TIF 7465 kb)

Additional file 8: PRISMA 2009 Checklist. (DOC 63 kb)

## Abbreviations

CDK1: Cyclin-dependent kinase1
CIs: Confidence intervals
DFS: Disease-free survival
EGFR: Epidermal growth factor receptor
HR: Hazard ratio
IHC: Immunohistochemistry
NSCLC: Non-small cell lung cancer
OR: Odds ratio
OS: Overall survival
SCLC: Small cell lung cancer
SQC: Squamous carcinoma
