# Supplementary figures and images for: Augmented expression of Ki-67 is correlated with clinicopathological characteristics and prognosis for lung cancer patients: an up-dated systematic review and meta-analysis with 108 studies and 14,732 patients
Source: Respir Res. 2018 Aug 13;19:150. doi: 10.1186/s12931-018-0843-7 (PMC6088431; doi:10.1186/s12931-018-0843-7)

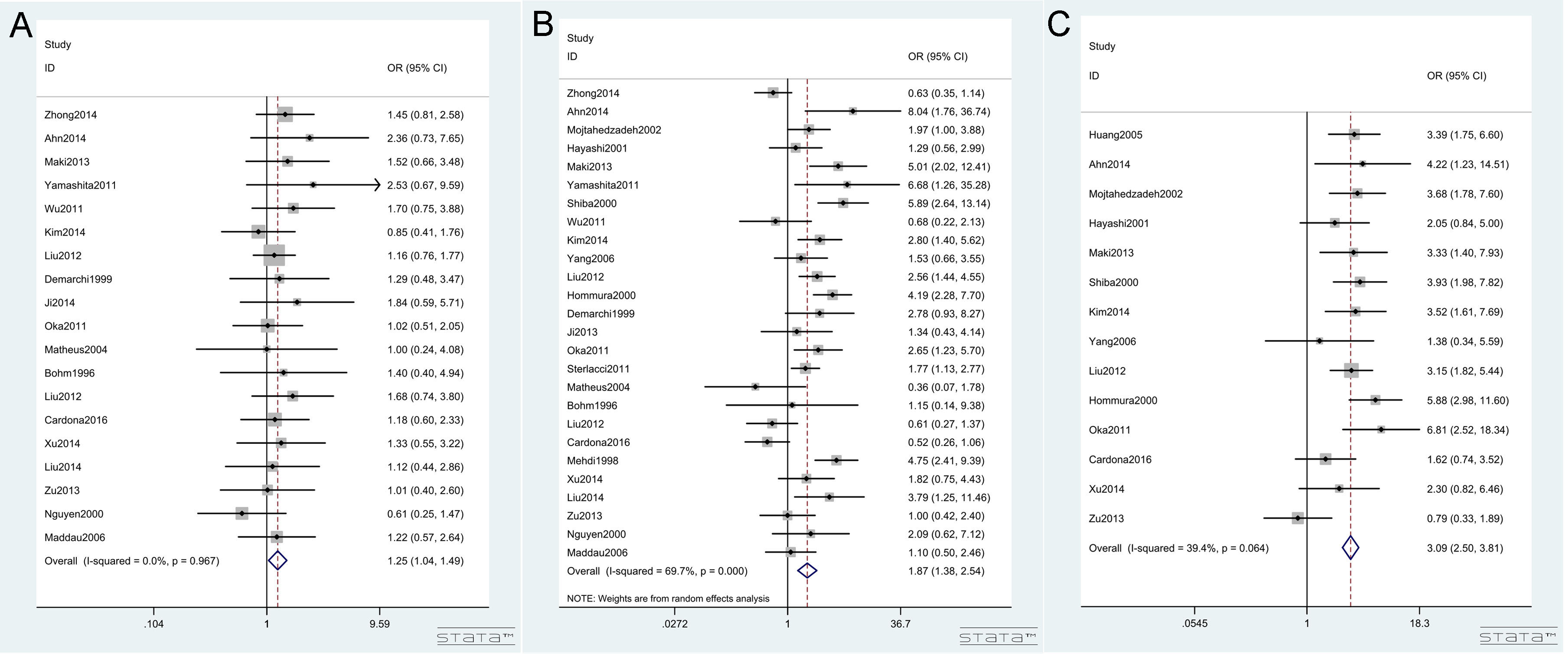

Supplement: Supplementary file 1 — Figure S1. Forest plots for the relationships between Ki-67 expression and clinicopathological features of patients with lung cancer. A. Age B. Gender C. Histological type. (TIF 4444 kb) [file 12931_2018_843_MOESM1_ESM.tif]

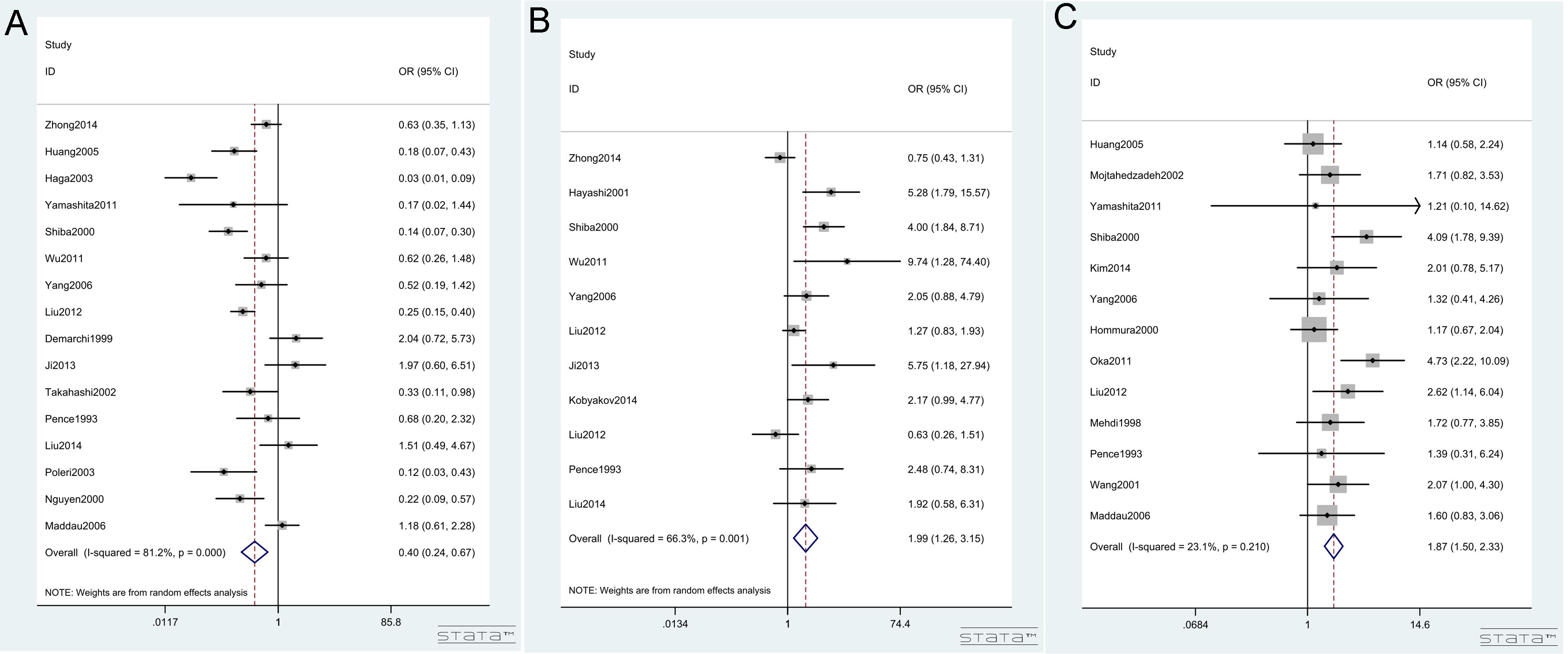

Supplement: Supplementary file 2 — Figure S2. Forest plots for the relationships between Ki-67 expression and clinicopathological features of patients with lung cancer. A. Differentiation B. Pathologic stage C. Tumor size. (TIF 3759 kb) [file 12931_2018_843_MOESM2_ESM.tif]

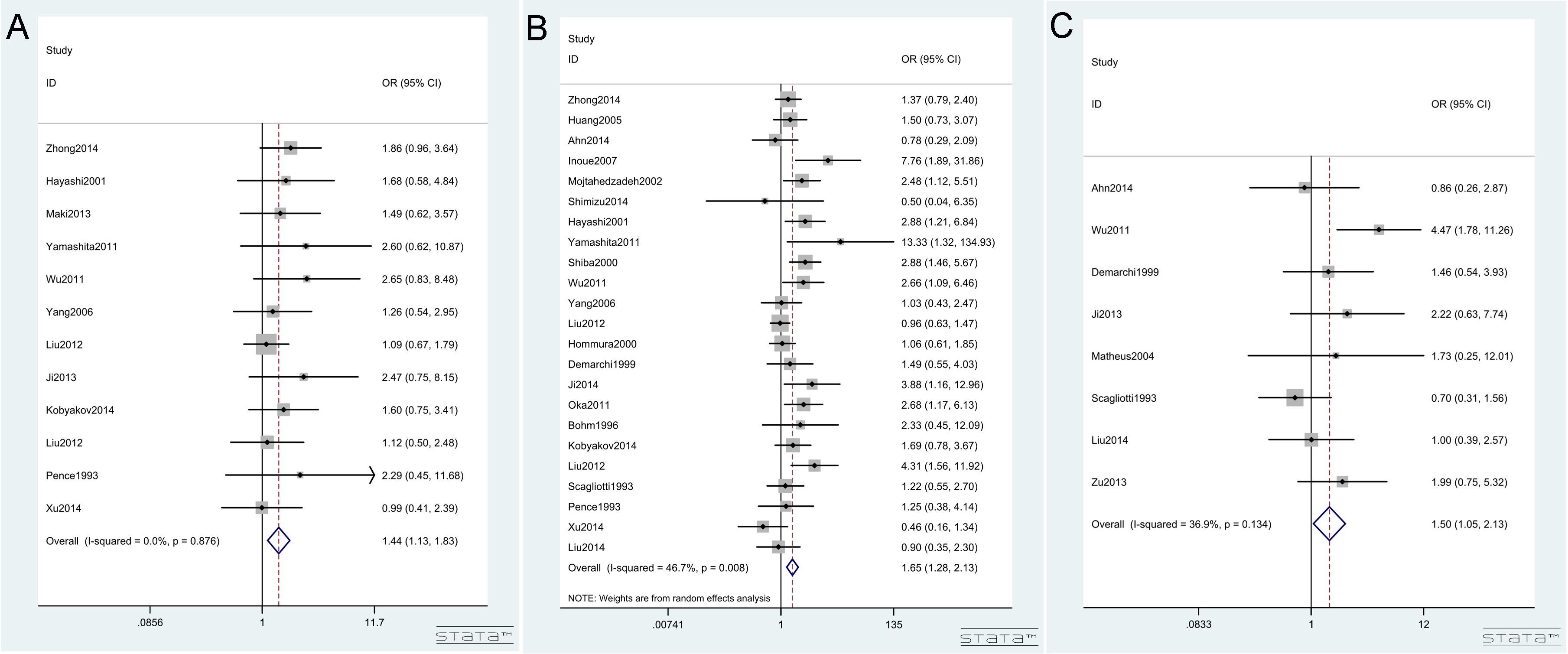

Supplement: Supplementary file 3 — Figure S3. Forest plots for the relationships between Ki-67 expression and clinicopathological features of patients with lung cancer. A. Lymph node B. TNM stage C. Smoking. (TIF 3186 kb) [file 12931_2018_843_MOESM3_ESM.tif]

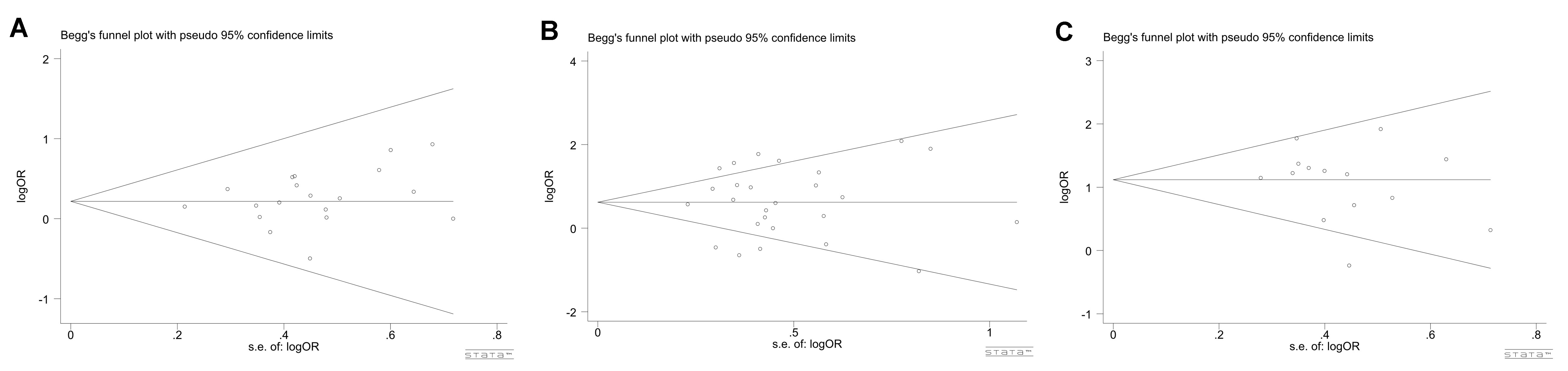

Supplement: Supplementary file 4 — Figure S4. Funnel plots for publication bias of clinicopathological features meta-analysis (A~C). A. Age B. Gender C. Histological type. (TIF 20061 kb) [file 12931_2018_843_MOESM4_ESM.tif]

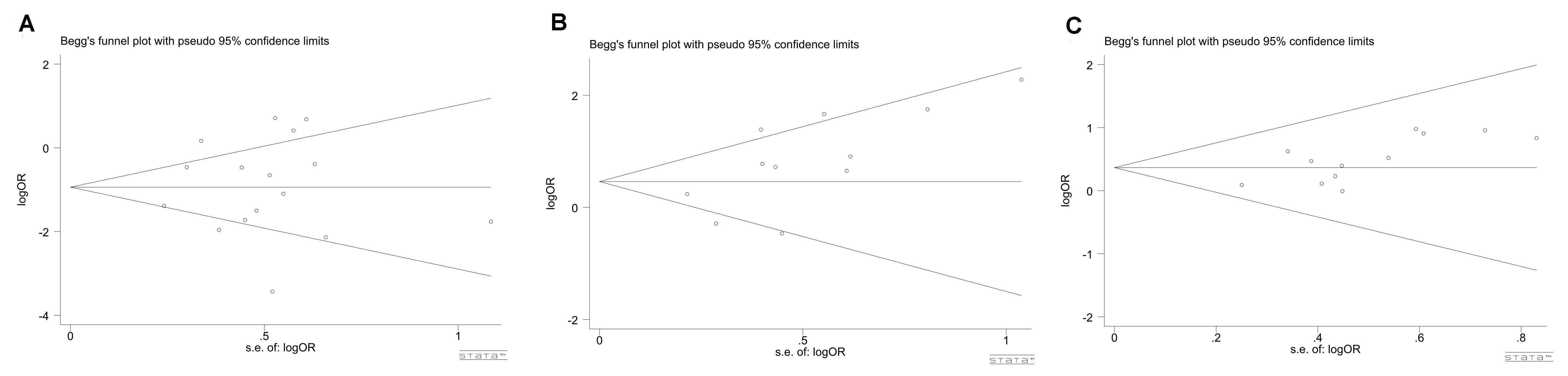

Supplement: Supplementary file 5 — Figure S5. Funnel plots for publication bias of clinicopathological features meta-analysis (D~F). A. Differentiation B. Pathologic stage C. Tumor size. (TIF 21452 kb) [file 12931_2018_843_MOESM5_ESM.tif]

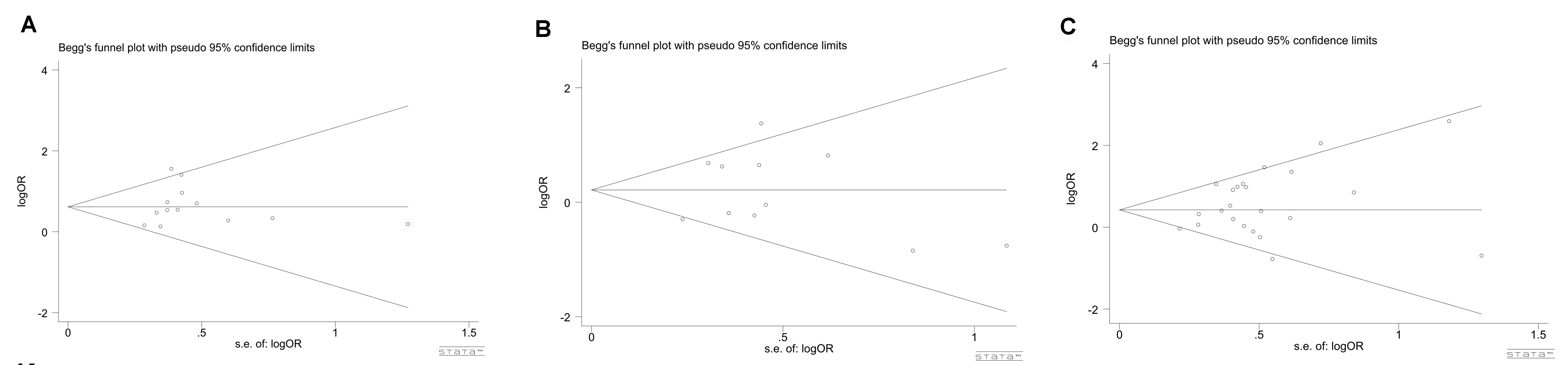

Supplement: Supplementary file 6 — Figure S6. Funnel plots for publication bias of clinicopathological features meta-analysis (G~J). A. Tumor stage B. Lymph node C. TNM stage. (TIF 21299 kb) [file 12931_2018_843_MOESM6_ESM.tif]

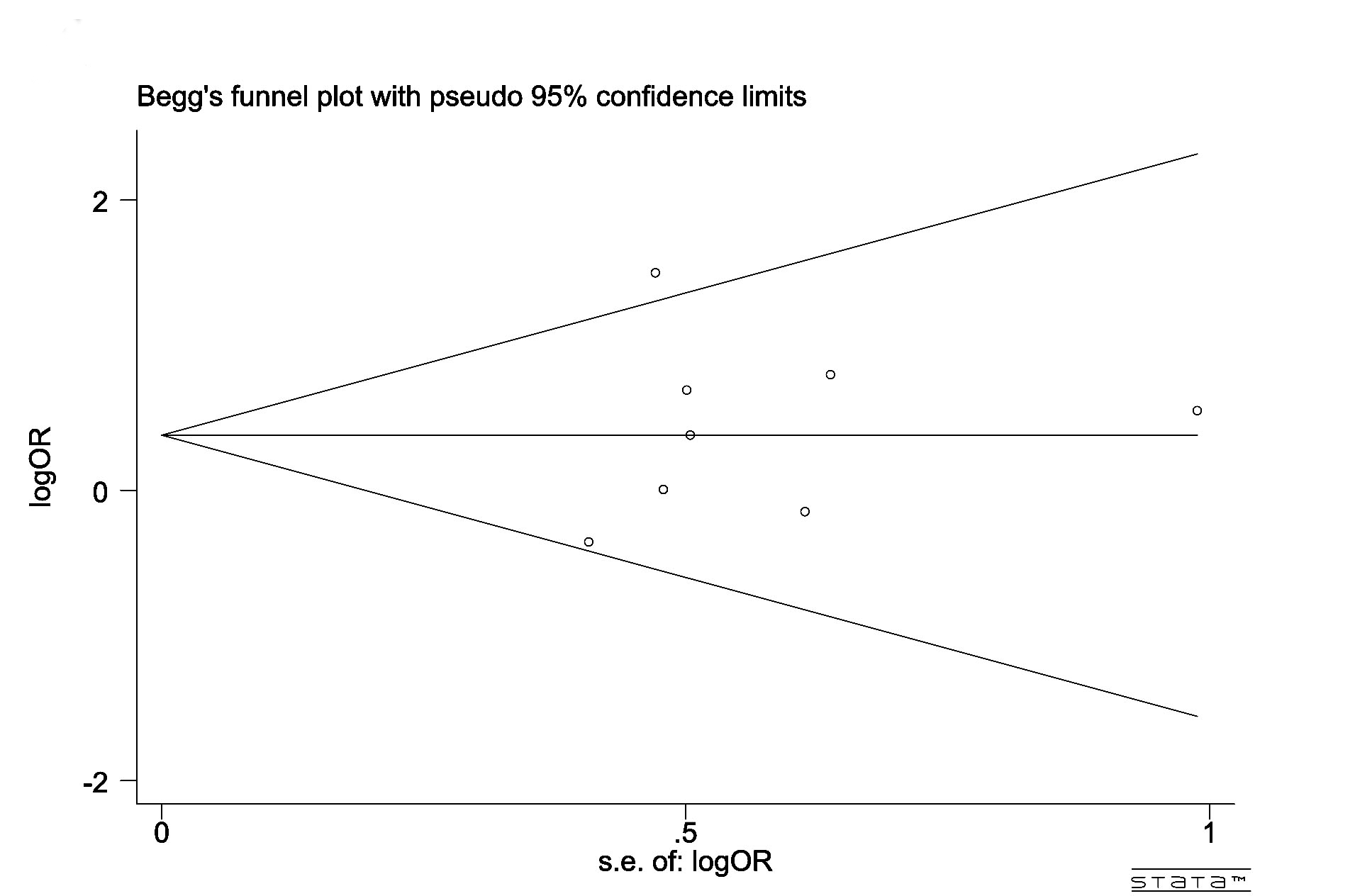

Supplement: Supplementary file 7 — Figure S7. Funnel plot for publication bias of clinicopathological features meta-analysis (Smoking status). (TIF 7465 kb) [file 12931_2018_843_MOESM7_ESM.tif]
